# Structural Health Monitoring Using a New Type of Distributed Fiber Optic Smart Textiles in Combination with Optical Frequency Domain Reflectometry (OFDR): Taking a Pedestrian Bridge as Case Study

**DOI:** 10.3390/s23031591

**Published:** 2023-02-01

**Authors:** Sabrina Abedin, Andres M. Biondi, Rui Wu, Lidan Cao, Xingwei Wang

**Affiliations:** Department of Electrical and Computer Engineering, University of Massachusetts Lowell, Lowell, MA 01854, USA

**Keywords:** distributed fiber optic sensors, structural health monitoring, dynamic strain measurements, smart textile, OFDR

## Abstract

Distributed fiber optic sensors (DFOS) have become a new method for continuously monitoring infrastructure status. However, the fiber’s fragility and the installation’s complexity are some of the main drawbacks of this monitoring approach. This paper aims to overcome this limitation by embedding a fiber optic sensor into a textile for a faster and easier installation process. To demonstrate its feasibility, the smart textile was installed on a pedestrian bridge at the University of Massachusetts Lowell. In addition, dynamic strain data were collected for two different years (2021 and 2022) using Optical Frequency Domain Reflectometry (OFDR) and compared, to determine the variability of the data after one year of installation. We determined that no significant change was observed in the response pattern, and the difference between the amplitude of both datasets was 14% (one person jumping on the bridge) and 43% (two people jumping) at the first frequency band. This result shows the proposed system’s functionality after one year of installation, as well as its potential use for traffic monitoring.

## 1. Introduction

Continuous monitoring of civil infrastructure is desirable for the detection of damage at the earliest opportunity, to ensure safety as well as economic purpose. The process of identifying such damage and monitoring the health of aerospace, civil, and mechanical engineering infrastructure is referred to as structural health monitoring (SHM). In this process, infrastructure is monitored for a continuous period and the measured results are compared to detect any abnormalities in the structure. Statistical analysis of the collected datasets allows the current state of the structure to be determined, from which necessary precautions can be taken if any damage or malfunctions are found. After an extreme event, such as an earthquake or unanticipated blast loading, SHM is helpful for rapid condition screening [1,2]. Moreover, for civil infrastructure such as bridges, continuous SHM is essential to ensure human safety. For this reason, several pieces of research are underway, looking at cost-effective, accurate and rapid measurement of the condition of any structure. In some conventional methods, electrical-based sensing systems such as strain gauges are used to monitor strain variations as part of the structural monitoring of railway infrastructure [3]. A wireless sensor network (WSN) was used for SHM installed on Golden Gate Bridge (GGB) [4] and ambient structural vibration was measured. However, this approach has limitations in handling heavy traffic and increasing packet size. Acoustic emission (AE) testing, another way to monitor civil infrastructure, is a nondestructive evaluation method for which MEMS sensors were proposed for AE detection [5].

However, for continuous structural monitoring, fiber optic sensors (FOS) are advantageous over other conventional methods, as they are immune to electromagnetic interference and can survive in extreme environments, such as radiation-hard and high temperatures [6,7,8]. Due to the capability of multiplexing, FOSs are suitable for long-range SHM, such as sewerage tunnels, buildings, dams, and bridges, to detect abnormalities or damage [9,10,11,12,13,14]. However, installing a single sensor at multiple distances provides only discrete information and adds an additional cost for every sensor element. If the monitored civil infrastructure is large, it will require more sensors throughout to obtain sensing information from the entire structure. A representation method of in-service performance has been presented [15], using a long-span cable-stayed railway–highway combined bridge as the case study. In this work, the authors collected distributed information from multiple types of sensing data to evaluate the real-time performance of the bridge and monitor its current condition. To overcome the limitations of installing multiple single sensors at multiple points, distributed fiber optics sensors (DFOS) have been developed for a complete picture of the current structural condition [16]. The beauty of DFOS is that the entire fiber becomes a sensor with multiple sensing locations, allowing the measurement of large amounts of strain, temperature, or vibration information for SHM.

The fragile nature of FOS, as well as the time-consuming installation process and labor costs, are some of the drawbacks of this approach. To ease the fiber handling and installation process, FOS have been embedded into textiles which provide full integration with the infrastructure [17,18,19]. These smart textiles play a growing role in developing an SHM system, as they can be installed on any structural shape and can reach through their curvatures, such as bridges, where it is difficult to install conventional sensing systems. They could also be used in monitoring strain changes of any composite substrates [20]. These kinds of textiles can be considered multifunctional or even “intelligent”, as they can sense various parameters such as temperature, strain, vibration, and any chemical substances by using the sensors installed on them [21]. For these advantages, this paper uses DFOS embedded in a textile to measure the strain variations with load changes, installing the sensor on a pedestrian bridge.

There are several interrogation methods used in DFOS, which can be classified as Raman, Brillouin, and Rayleigh distributed sensing. Raman backscattering is used for distributed temperature sensing, as the associated amplitudes of Stokes/anti-Stokes components depend on temperature. Brillouin backscattering is an inelastic scattering process using the analysis of the reflected frequency spectrum to measure both strain and temperature. Rayleigh scattering is an elastic process widely used in SHM [22]. Nowadays, Rayleigh-based Optical Frequency Domain Reflectometry (OFDR) interrogation systems are frequently used to measure both temperature and strain with high spatial resolution [23,24].

In this approach, the textile-embedded DFOS system provides not only distributed information of the strain but also full integrity to install it on the pedestrian bridge. This process reduces the labor cost and eases the installation process by reducing the cable requirements of conventional sensor installation based on strain gauges. In this installed sensor, a single fiber optic cable senses the whole sensing area. However, in a strain gauge-based sensing system, every strain gauge requires at least two wires, complicating the system. The OFDR interrogation method is used to measure the dynamic response of the sensor. To the best of our knowledge, this is a novel approach for measuring the dynamic response of a pedestrian bridge based on DFOS embedded into textiles using OFDR. Through this approach, the proposed sensor can also sense the load variations of the bridge as strain changes. To validate the sensor’s response over time, a comparison of the sensor’s response over two consecutive years is presented. As the installed sensor was kept in the pedestrian bridge during this time, it can be said that it did not hinder the regular activity of the pedestrian bridge due to the flexibility and rigidity of the installation process on the textile. Another advantage of our approach is that it overcomes one of SHM’s challenges, which is the theoretical approach to practical implementation. Installation on a pedestrian bridge and being able to record the dynamic response for two consecutive years ensures that the implemented system is suitable for the practical application of SHM.

The paper is organized as follows: Section 2 describes the DFOS system that was used to record the dynamic responses, as well as illustrating the fabrication and installation process of the textile-embedded DFOS on the pedestrian bridge. In Section 3, recorded responses of 2021 and 2022 tests are analyzed and then compared to validate the sensor’s efficacy. Section 4 discusses the results, reasons for signal degradation, and their possible solutions as a future approach. Finally, this paper is concluded in Section 5.

## 2. Distributed Fiber Optic Sensing System

### 2.1. OFDR Interrogation Method

In this work, which proposed to collect the dynamic response in terms of strain variation, the Optical Frequency Domain Reflectometry (OFDR) interrogation method was used. The Rayleigh scattering-based OFDR method is advantageous due to its high spatial resolution (millimeters) and large dynamic range [25]. Eickhoff et al. first presented the OFDR method based on Rayleigh backscattering of an optical fiber in 1981 [26]. Rayleigh scattering is generated by random fluctuations of the refractive index in the core. These random fluctuations along the fiber core can be considered Bragg grating, with random variation of amplitude and phase.

Any change as strain or temperature variations on the fiber under test can result in a local reflected spectral shift. This is obtained from the cross-correlation between two Rayleigh backscattering spectra measurements, the first a measured backscattered pattern and the second a reference fingerprint. From this approach, changes in distributed sensing parameters such as strain, temperature, and vibration can be measured from the spectral shift [25,27]. The following equation represents the relationship between the spectral shift ∆ν, the temperature variation ∆T, and the strain variation ∆ε:

∆ν = *K*_T_ ∆T + *K*_ε_∆ε (1)

where *K*_T_ and *K*_ε_ are the temperature and strain-sensitive coefficients, respectively.

This paper uses this interrogation method to record the dynamic response of a pedestrian bridge to strain variation.

### 2.2. Fabrication of Optical Sensor Embedded in Smart Textiles

**Figure 1:**
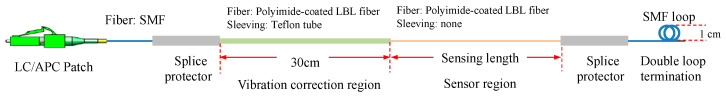
Schematic of an OFDR strain sensor.

The OFDR strain sensor was fabricated in our lab; the schematic is shown in Figure 1. A polyimide-coated, low bend loss (LBL) fiber was spliced with an LC/APC connector as the sensing section and a Teflon tube (28-gauge LW tubing, Zeus) was used as the vibration correction region. The Teflon sleeving was 30 cm long and spliced with the first splicing protector after the fiber connector. The Teflon sleeving could effectively reduce the vibration conducted to the connector. The other end of the sensing section was spliced with a single mode optical fiber (SMF) and created a double loop knot as the termination. The termination was a key structure to the OFDR sensors, since the machine was a marker to recognize the end of the sensor. This section requires normal SMF, as the banding loop creates a significant drop in light reflection, which the OFDR demodulator depends upon to recognize the end of the sensors.

**Figure 2:**
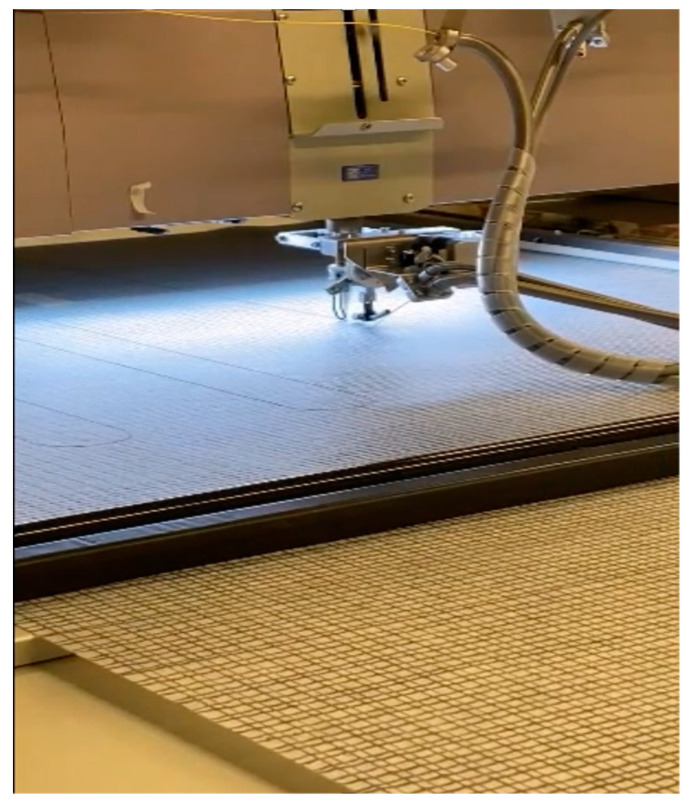
Picture of an embroidery machine embedding a fiber sensor into a textile.

We worked with Saint-Gobain to embed the OFDR sensor into the textile. Using the embroidering machine, a method was developed to achieve the embedding of different structural designs into the textile. This method provides enormous potential to combine various fiber sensor designs and flexible structures with textiles. Figure 2 shows the embroidery machine embedding fiber into the textile, similar to the weaving process.

**Figure 3:**
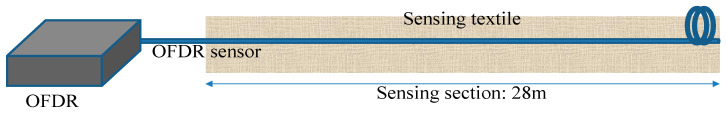
OFDR sensing textile schematic.

Figure 3 shows the optical pattern design of the sensing textile. The fiber was embedded into a cost-effective reinforcing fabric manufactured by Saint-Gobain (XP414 laid scrim, Northborough, MA, USA), made by chemically bonding continuous filament yarns in an open mesh construction. One straight optical fiber measuring longitudinal strain was distributed across the width of the textile. The length of the sensing section was 28 m. The optical fiber was connected to OFDR (ODiSI, Luna, Roanoke, VI, USA) to collect strain data. An advantage of this sensing textile was its capability to collect the spatial distribution of Rayleigh scattering on the sensing fiber, then reconstruct it to strain distribution. Spatial resolution of less than 1 mm can be achieved, according to the different parameters of machine settings.

### 2.3. Installation of the Sensor in the Pedestrian Bridge

**Figure 4:**
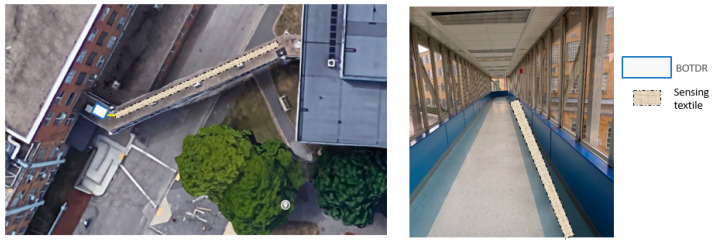
Location of installation.

The sensing textile prototype was planned to be installed on one side of the floor, as shown in Figure 4.

The sensing textile was installed following the procedure listed below.

**Figure 5:**
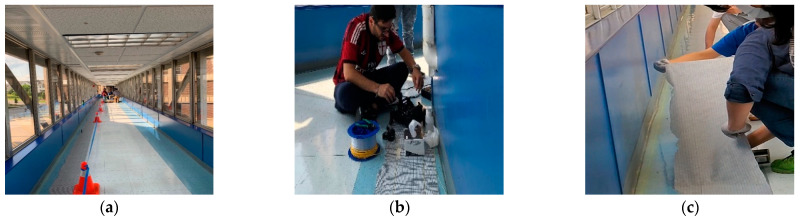
(**a**) Placing the sensing textile. (**b**) Extending the sensing textile to an optical fiber spool. (**c**) Applying 3M Hi-Strength 90 spray adhesive between the floor and the sensing textile.

First, the sensing textile was placed at the designated location on the floor, as shown in Figure 5a. With the optical fiber side facing down, the integrity of the fibers was then checked using an Optical Time Domain Reflectometer (OTDR) to make sure they were working well, and extended the sensing textile to an optical fiber spool (Figure 5b). The 4-ft sensing textile was lifted from the floor and 3M Hi-Strength 90 spray adhesive was applied on both, the floor and the underside of the sensing textile, as shown in Figure 5c. After that, the sensing textile was placed back on the floor and the air bubbles were removed with our hands and putty knives. These steps were repeated on every 4-ft of the sensing textile until the entire sensing textile was glued to the floor.

## 3. Results

### 3.1. Dynamic Response of 2022 Test

To collect the dynamic response of the pedestrian bridge at UML, a field test was conducted in August 2022. To collect the dynamic response, he OFDR interrogator system was connected with one end of the installed distributed fiber optic sensor, using the approach presented in Figure 3. The dynamic response was collected first when one person jumped on the bridge and then when two people jumped. As the installed sensor was designed as an OFDR strain sensor following the schematic shown in Figure 1, the dynamic response was collected to measure the strain variation with different loads.

**Figure 6:**
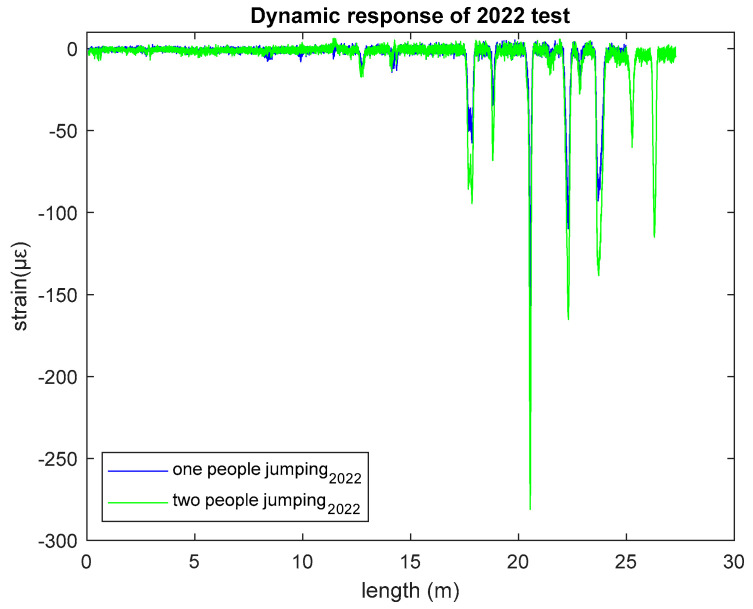
Dynamic response of 2022 test.

Figure 6 depicts the strain variation along the whole fiber optic sensor length (approximately 28 m). In the response plot, the variation mostly occurs from the center to the end of the fiber, as the individuals jumping stood near the center. Figure 6 shows that two people jumping creates greater strain value (maximum −281 µε) than one person jumping (maximum −176.9 µε). This data also confirms that our textile-embedded sensor can sense strain change according to the load variation. The greater the load, the greater strain change occurs.

### 3.2. Dynamic Response of 2021 Test

**Figure 7:**
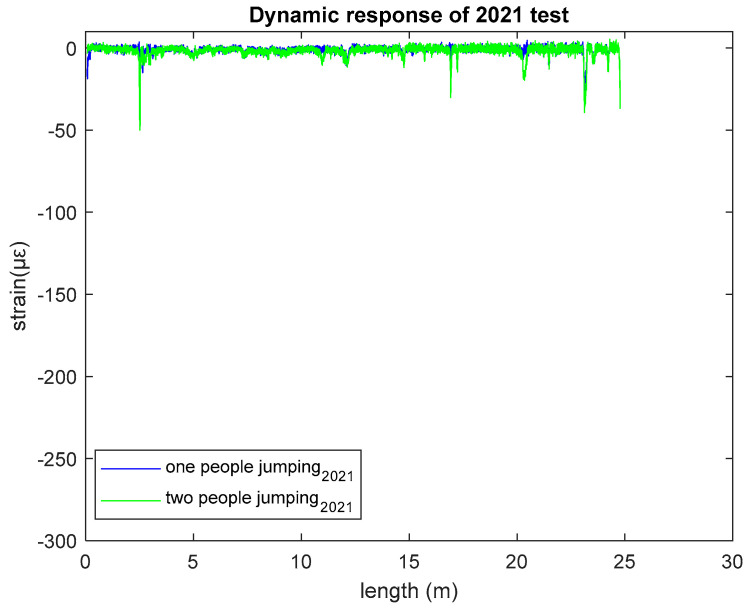
Dynamic response of 2021 test.

In May 2021, another field test was conducted to collect the dynamic response of the UML pedestrian bridge. Using the same approach as described for the 2022 test, the dynamic response was recorded for both one and two people jumping, displayed in Figure 7.

From Figure 7, as with the 2022 test results, it was found that two people jumping provides greater strain value (maximum −50.1 µε) than one person jumping (maximum −38.5 µε). This year’s test results also showed the same relationship between load and strain as found in 2022.

### 3.3. Comparison of Two Years of Test Results

**Figure 8:**
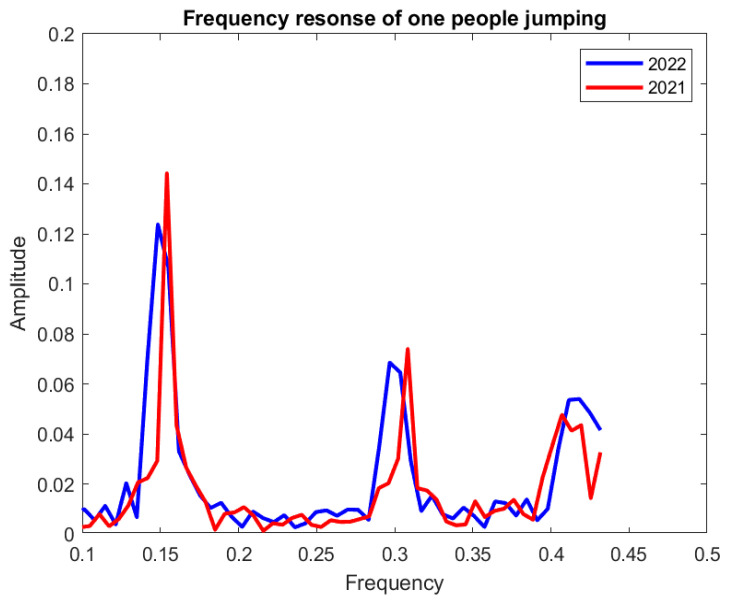
Comparison of two years’ sensor response for one person jumping.

**Figure 9:**
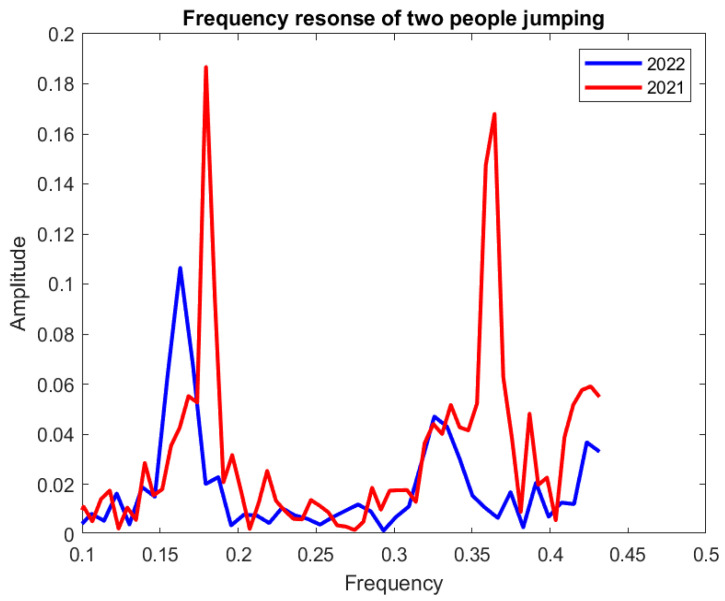
Comparison of two years’ sensor response for two people jumping.

To validate the sensor response and to ensure its durability, comparisons of the sensor’s response for the two consecutive years of test results are presented in Figure 8 and Figure 9 in terms of frequency domain analysis.

Figure 8 shows that, for one person jumping, the signal amplitude changes from 0.144 to 0.124 at the first frequency band and 0.074 to 0.068 at the second frequency band in the 2021 and 2022 test results, respectively. This signal changing for two people jumping is depicted in Figure 9 for both test years. In this case, at the first frequency band, the signal amplitude changes from 0.187 to 0.106, and at the second frequency band from 0.168 to 0.047, for 2021 and 2022, respectively.

## 4. Discussion

From Figure 8 and Figure 9, the 2022 test results showed a good agreement with the 2021 test results by exhibiting a similar system pattern, which demonstrates the sensor’s functionality after a year. It also shows that the sensor’s sensing capability was not interrupted by the regular activities of a pedestrian bridge. The only degradation is in the amplitude of 2022 test results versus 2021, which is calculated as around 14% for one person jumping and 43% for two people jumping at the first frequency band.

The degradation in amplitude may occur for several reasons. One is that the sensing textile is not completely flat against the bridge, as it was installed a few years ago and there may be some air bubbles which can degrade the sensor’s response. The surrounding environment (temperature) may also affect the collected responses. As part of temperature calibration, we calculated the temperature coefficient for the same proposed textile-embedded fiber in our lab and found the temperature coefficient value to be 0.99 MHz/°C for the −10 °C to 40 °C temperature range and 0.63 MHz/°C for 40 °C to 80 °C. Besides these reasons, the variation in load—in our case, the weight of the people—was also not constant between these two years. Another reason is the synchronization of the two people when they jumped. These reasons for amplitude degradation are the same for the second frequency band.

To improve these signal degradation problems, for future work to install any DFOS embedded in the textile, care will be taken to keep the textile as flat as possible to the bridge for long-term monitoring. This will prevent any air bubbles forming between the textile and the bridge a few years post-installation. Epoxy can be used to glue the protection mat compactly to the floor. If the sensor can be kept flat to the bridge, preventing air bubbles, it is expected that the signal degradation problem for long-term monitoring can be improved. To calculate the impact of temperature, we will install another fiber in our next installation, to be used for measuring temperature variations.

However, the similar system response ensures not only the sensor’s durability but also its robustness to detect the dynamic response of any pedestrian bridge using DFOS.

## 5. Conclusions

In this paper, two approaches are presented to contribute to structural health monitoring. One approach is a distributed fiber optic sensor embedded in textile which will reduce the cost of installing a single sensor at multiple points and which provides the integrity to install the sensor in different types of civil infrastructure. Due to its distributed nature, distributed information can be gathered, such as strain variation along the whole fiber length, which is useful for monitoring large structures and providing a complete picture of the structural condition. This proposed sensing system also has the potential for continuous monitoring if it is required by the end user. Another approach is to measure dynamic response using an OFDR interrogator, which will help to monitor the traffic as well as load variations of any bridge.

This paper explores the dynamic response as strain variations with load change. Our presented textile-embedded DFOS, with a sensing length of 28 m, was installed in a pedestrian bridge over a year without interrupting the regular activity of the bridge. To record dynamic response, datasets of one and two people jumping were measured for two consecutive years (2021 and 2022). After comparing the two years’ results, the sensor’s response showed good agreement with the previous year’s response. Although degradation of amplitude was found in 2022 than in 2021, the similar system response pattern confirmed that the installed sensor is functioning after one year. This ensures the durability and effectiveness of our implemented textile-embedded DFOS using OFDR.

## Data Availability

Not applicable.
